# An In-Depth Look at Fonni’s Dog Behavior under Different Outdoor Conditions

**DOI:** 10.3390/ani14050678

**Published:** 2024-02-21

**Authors:** Raffaella Cocco, Sara Sechi, Claudia Giannetto, Maria Rizzo, Giuseppe Piccione, Francesca Arfuso

**Affiliations:** 1 Department of Veterinary Medicine, University of Sassari, 07100 Sassari, Italy; rafco@uniss.it (R.C.); sarasechilavoro@tiscali.it (S.S.); 2 Department of Veterinary Sciences, University of Messina, 98168 Messina, Italy; rizzom@unime.it (M.R.); gpiccione@unime.it (G.P.); farfuso@unime.it (F.A.)

**Keywords:** behavior features, free-ranging dogs, Fonni’s Dog, kennel, body language, ethogram

## Abstract

**Simple Summary:**

In this study, the common social and communicative behaviors of the Fonni’s Dog maintained under different outdoor conditions were investigated in order to improve the scant knowledge currently available on the behavior features of this dog breed. The preliminary results obtained here show that both dogs confined to kennels and free-ranging ones showed a strong collaborative motivation with the owner. This indicates a behavior consistent with the attitude of the Fonni’s Dog.

**Abstract:**

This study aimed to investigate the common social and communicative behaviors of the Fonni’s Dog under different outdoor conditions. For this study, 70 adult dogs (3–7 years; 32 intact males, 38 intact females) belonging to the Fonni’s breed were used. A total of 35 dogs were kept in kennels and 35 were free-ranging dogs in their sheep/goat livestock units. A behavioral repertoire was adapted from the literature and an ethogram was filled in for each dog. All dogs were evaluated in the presence of the owner. Fisher’s exact test, following Bonferroni’s correction, was used to test possible differences in the categorical variables (presence or absence of the behavior) between free-ranging dogs and dogs kept in kennels. The study revealed that several categories of the dogs’ body language were associated with the management condition. However, the breed motivations (guarding and defense of the territory) were satisfied both in kennel and in the animals who were free in the property. The current study suggests a good behavioral balance of the Fonni’s Dogs which could be attributed to correct communication between dogs and owners.

## 1. Introduction

**Figure 1:**
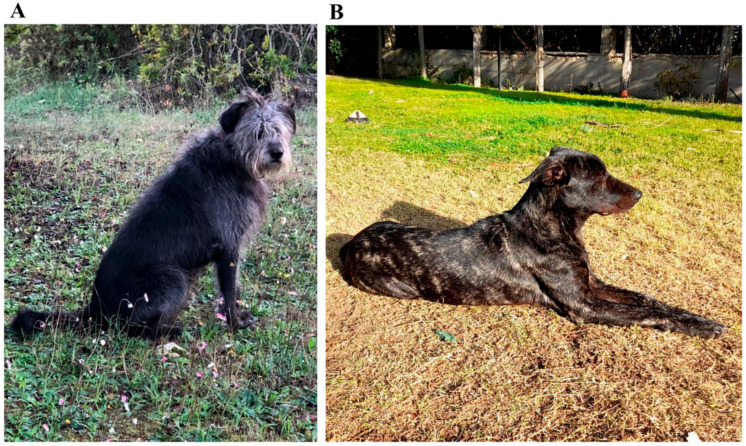
The rough-coated (**A**) and smooth-coated (**B**) varieties of Fonni’s Dogs.

The Fonni’s Dog is a livestock and property guardian breed originating from the region surrounding the city of Fonni on the island of Sardinia. International breed recognition of the Fonni’s Dog is being pursued by a dedicated group of breeders and enthusiasts (http://www.canefonnese.it/ accessed on 10 April 2022), with a goal of preserving the distinct heritage of this remarkable breed. The morphological commonalities of the Fonni’s Dog have been previously characterized, showing that they are consistent with features of a true-breeding population [1]. This hypothesis has been supported by the investigation of a limited number of microsatellite markers [1]. The rough-coated and smooth-coated varieties are both recognized in Fonni’s Dogs (Figure 1).

The Fonni’s Dog is traditionally inserted in a rather isolated geographical context, consisting of the mountainous and innermost region of the Italian island. The genetic history of the Fonni’s Dog parallels demographic events in local human populations. Though it is still matter of debate, some traces of lithic industry would suggest the presence of Paleolithic man in Sardinia, and, in this case the presence of the domestic dog would be theoretically conceivable, probably as an auxiliary for hunting activity [1,2,3]. However, the presence of the dog in Sardinia has been demonstrated starting from the Neolithic Age, when people began to live in permanent settlements and practice agriculture and animal breeding [4,5,6]. Archeozoological studies carried out on osteological remains highlighted the role of Sardinian prehistoric dogs in ancient communities [6]. The discovery of dog remains during archeological excavation of Nuragic settlements (II mill.–II cent. BC) permitted the reconstruction of the morphological features of Bronze Age dogs coinciding with the Nuragic Age in Sardinia. Nuragic dogs were mesocephalic, and characterized by the presence of prominent muscle insertions; the estimation of their shoulder height is about 51–55 cm. The Fonni’s Dog’s ancestors were probably in that canine population and it is described with rough- and smooth-coated varieties. The unifying features of the breed include a characteristically intense facial expression and instinctive propensity toward guarding behaviors and wariness of strangers. Historical accounts portray dogs fitting this description residing in Fonni (Sardinia) and the surrounding regions in the mid- to late-19th century [7,8,9]. It has been suggested that morphological commonalities of the Fonni’s Dog are consistent with features of a true-breeding population as supported by a limited number of microsatellite markers [1]. Moreover, in a whole-genome-sequence study, the genomic architecture of the Fonni’s Dog has been described [10]; the propensity for this breed to display genomic characteristics equivalent to those of established and acknowledged breeds has been demonstrated, suggesting that the geographic isolation and behavior-driven selection function produce unique breed populations [10]. The personality of animals is identified with individual consistency in behavioral reactivity to stimuli and situations [11,12,13,14,15,16,17,18,19,20].

The study of the behavioral characteristics of Fonni’s Dogs is of great interest if placed in relation to the particular breeding methods used for the selection of the canine specimens and with the role of watchdogs of the flock or of the property that these animals have always held. In the past, there was a single breeding methodology for the selection and training of these dogs, which consisted in raising the puppies without having contact with humans. These were then kept in holes dug in the ground and covered with branches (sensory deprivation syndrome). Some puppies, especially white-colored females, were fed sheep’s milk directly sucked from the animal’s udder, so that a bond is created between sheep and dog and the concept of “species” to be defended at all costs is reinforced.

A single animal, if well-trained, was able to watch and lead an entire flock, and to protect it from predators such as foxes and/or stray dogs. In order to encourage the marked attitude of these dogs for watching and defending animals and territory, they were trained, from an early age, to chase and attack a puppet with a stomach full of sheep’s blood tied around the neck. They were praised and amply rewarded by the owner every time they attacked the puppet in the neck. Nowadays, the most common breeding practices include: (i) Animals allocated in kennels, (ii) Animals free on the property (they have the role of watchdogs and sometimes of hunting dogs), (iii) As pets (this group includes dogs living in apartments) [20]. Though considerable effort has gone into decoding the genetic basis of morphologic traits that vary within dog populations, the challenge of identifying behavioral features characterizing the Fonni’s Dog is largely unmet. It could be hypothesized that management conditions and/or the presence of strangers could influence the behavior of the animal. Dogs kept in kennels might be more fearful of humans (for shutting them into kennels), be more aggressive toward humans (resisting being locked into the kennel), be more dominant, or desire freedom to roam or run more than free-ranging dogs. All these conditions could modify the behavior of dogs and, therefore, their body language. The purpose of the current study was to investigate the common social and communicative behaviors of the Fonni’s Dog maintained under different outdoor conditions in order to improve the scant knowledge currently available on the behavior features of this dog breed.

## 2. Materials and Methods

For this study, 70 adult dogs (3–7 years; 32 intact males, 38 intact females) belonging to Fonni’s Dog breed were used. All dog owners signed an informed consent before participating in the study. All operative procedures, housing, and animal care reported below were carried out in accordance with the standards recommended by Directive 2010/63/EU [21] on the protection of animals used for scientific purposes. The recommendations of the ARRIVE guidelines were also consulted [22]. The study was carried out in various farms in northern Sardinia (Italy) during the spring 2022 (mean ambient temperature 11.7 °C ± 2.5 °C, mean relative humidity 78% ± 1.9%). At the beginning of the study (enrollment of each dog) the health status of each dog was checked. In particular, accurate clinical examinations and laboratory tests (hematological and biochemical profiles) were carried out on each of them to exclude pathologies as possible primary or secondary cause of behavioral problems. Moreover, their health status was evaluated based on behavior, rectal temperature, heart rate, respiratory profile, cough, nasal discharge, ocular discharge, appetite, and fecal consistency. The dogs were clinically healthy, free from external and internal parasites, and in good nutritional condition.

In order to study the morphological characteristics, each dog was measured with the aid of professional tools: kinometer, meter, 30 cm caliber, as previously described [1]. The measurements were carried out by the same operator, different from the one who took care of the measurements on behavioral characteristics. The phenotypic characteristics of enrolled dogs were within the standard of the breed established by the National Italian Dog Breeding Organization, Milan, Italy (https://www.enci.it/libro-genealogico/razze/cane-fonnese, accessed on 4 April 2022), and in agreement with previous observations [1].

The dogs enrolled in the study were divided into two groups according to their management conditions. Briefly, dogs who, prior to the study, lived in kennels were assigned to the kennel condition and dogs who, prior to the study, did not live in kennels were assigned to the free-ranging condition. Specifically, 35 dogs (*n* = 21 intact females and 14 intact males; 5 ± 2 years old) from different farms (1 dog per farm) were kept in galvanized steel kennels comprising two sections: an indoor section (3 m × 2 m × 1.60 m) and an outdoor section (3 m × 4 m × 1.60 m), under natural photoperiod and a natural environmental temperature. These dogs lived free on their respective farms but they were used to being kept in kennels. Precisely one month before the study, the dogs were kept in kennels daily for at least 10 h.

A total of 35 were free-ranging dogs (*n* = 17 intact females and 18 intact males; 5 ± 2 years old) in their sheep/goat livestock units (1 dog per farm), under natural environmental conditions.

Each dog was away from human contact with the exception of owner, and they were provided, by the owner, with the same commercial adult dog maintenance food (crude protein 25.00%, fats 13.00%, crude fiber 2.50%, ash 8.00%, calcium 1.46%, phosphorus 1.16%), according to industry recommendations. Food was given twice a day, at 6:00 in the morning and at 17:00 in the evening. Water was available ad libitum.

A single male well-trained behaviorist researcher observed and videotaped each dog in its environment. Each dog was observed in its environment (kennel dogs in their kennel and free-ranging dogs on the farm).

One 30 min observation of each dog was performed. The videos were subsequently reviewed by a team of 4 veterinarians in order to test the objectivity of the surveys in the field.

Dogs of both groups were allowed to freely explore the environment while the owner and the researcher sat on chairs. Specifically, the kennel dogs were allowed to freely explore the kennel, whereas the free-ranging dogs were allowed to freely explore the farm during the observation. 

The dogs and the owners were free to interact, while the researcher remained as neutral as possible: they stayed still, did not talk to the dogs, pet them, make eye contact with them, or punish them. Moreover, the dogs were evaluated free and in kennels in presence of the owner. A behavioral repertoire was adapted from the literature to record the stress-related behaviors of dogs and an ethogram was filled in for each dog (Table 1).

To test the categorical variables (presence or absence of the behavior) for goodness of fit, homogeneity, and independence, and considering the non-random associations between categorical variables, the Fisher’s exact test with Bonferroni’s correction was used for each recorded behavior category. *p*-values < 0.025 for category with 2 observations (body expressions and excretions), *p* < 0.017 for category with 3 observations (ear appearances and vocalization), *p*-values < 0.013 for category with 4 observations (mouth expressions and tail language), and *p*-values < 0.010 for the category with 5 observations (contact) were considered statistically significant after Bonferroni’s correction. All data were analyzed using the STATISTICA 8 (Stat Soft Inc., Tulsa, OK, USA) statistical software.

## 3. Results

**Figure 2:**
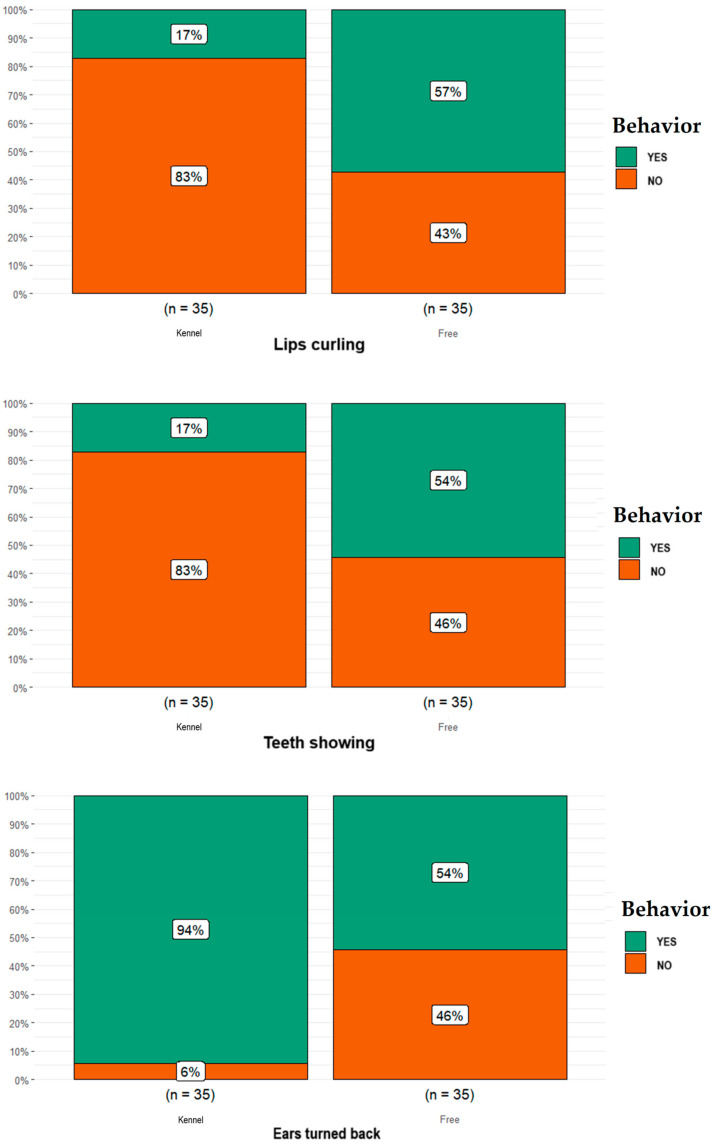
Differences obtained in the mouth expressions category (i.e., lip curling against researcher, teeth showing against researcher) and for the ear appearances category (i.e., dogs having ears turned back) between the free-ranging Fonni’s Dogs and those kept in kennels.

**Figure 3:**
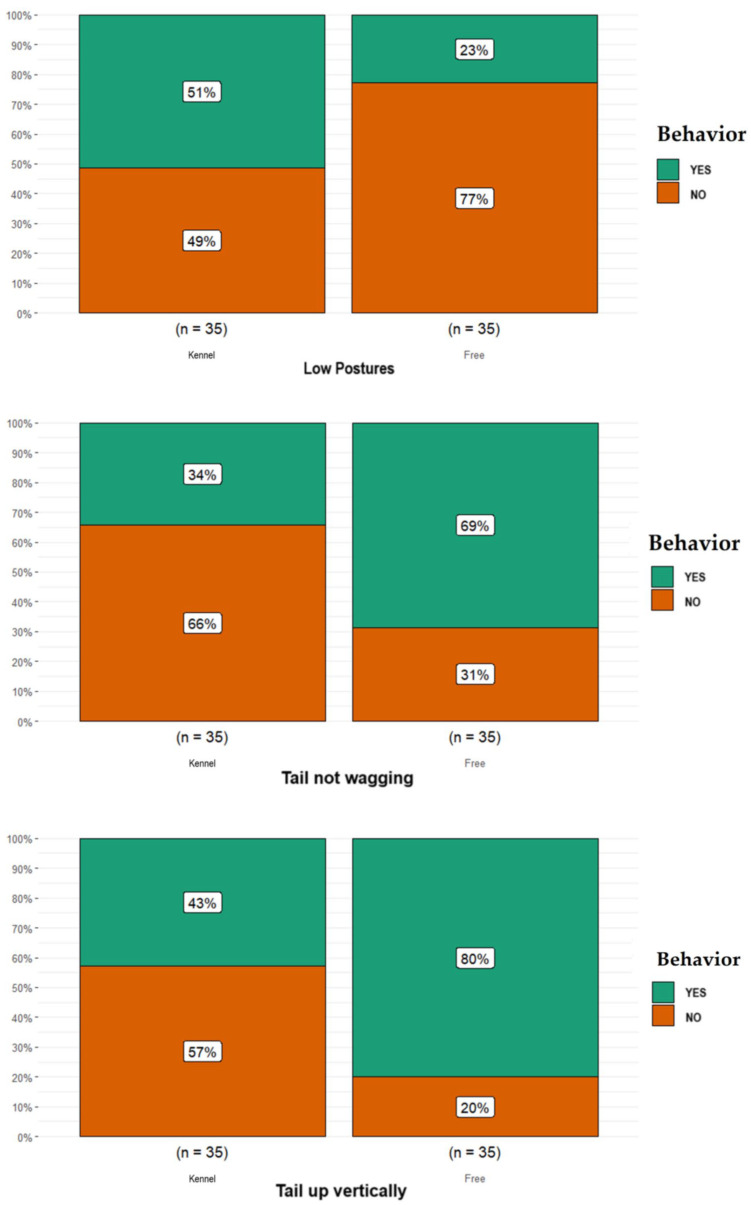
Differences obtained in the body expressions category (i.e., low postures) and in the tail language category (i.e., not tail-wagging, tail up almost vertically) between the free-ranging Fonni’s Dogs and those kept in kennels.

Statistical analysis showed many significant differences in considered behaviors between free-ranging dogs and dogs kept in kennels. In particular, as shown in Figure 2, within the mouth expressions category, lip curling against researcher was more frequent in free-ranging dogs (*n* = 20) than dogs kept in kennels (*n* = 6; *p* = 0.001); the proportion of free-ranging dogs reported as having teeth showing against researcher (*n* = 19) was higher than that for dog kept in kennels (*n* = 6; *p* = 0.002). Within the ear appearances category, the proportion of free-ranging dogs reported as having ears turned back was lower (*n* = 19) than that for the proportion of dogs kept in kennels (*n* = 33; *p* = 0.0002; Figure 2). Regarding the body expressions category, as shown in Figure 3, the proportion of free-ranging dogs reported having low postures (*n* = 8) was lower than that for dogs kept in kennels (*n* = 18; *p* = 0.024). Concerning the tail language category, the proportion of free-ranging dogs reported as not having tail-wagging (*n* = 24) was higher than that for dogs kept in kennels (*n* = 12; *p* = 0.008); the proportion of free-ranging dogs reported as having the tail up almost vertically (*n* = 28) was higher than that for dogs kept in kennels (*n* = 15; *p* = 0.003, Figure 3).

**Figure 4:**
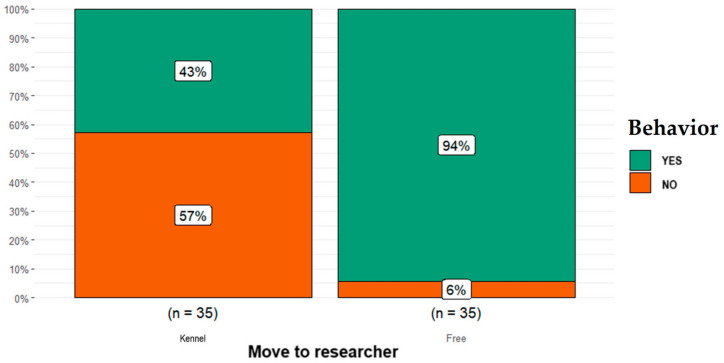
Differences obtained in the contact category (i.e., dog moving toward researcher) between the free-ranging Fonni’s Dogs and those kept in kennels.

As shown in Figure 4, regarding the contact category, the proportion of free-ranging dogs reported as having moved toward researcher (*n* = 33) was higher than that for dogs kept in kennels (*n* = 15; *p* = 0.0001).

**Figure 5:**
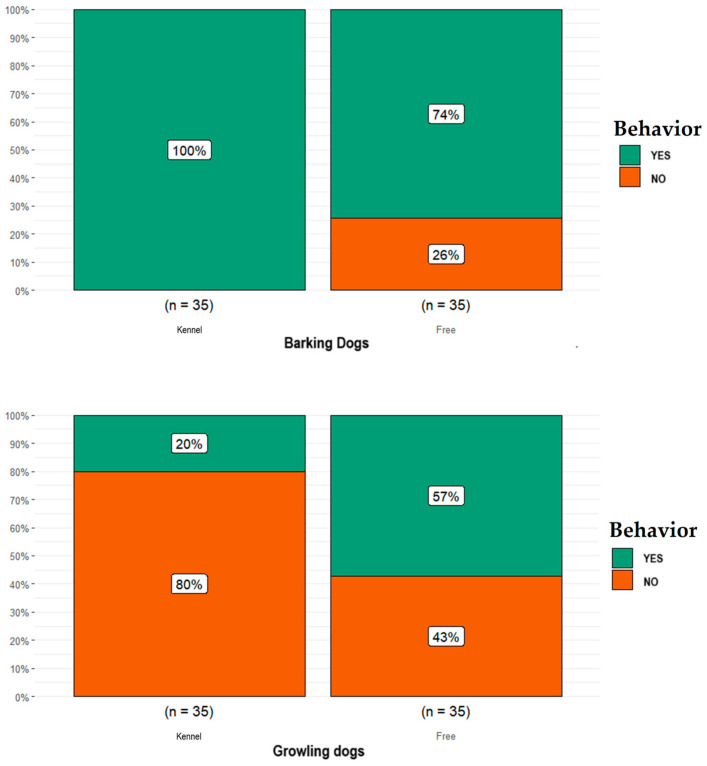
Differences obtained in the vocalization category (i.e., barking dogs, growling dogs) between the free-ranging Fonni’s Dogs and those kept in kennels.

When data were analyzed according to vocalization category, the proportion of free-ranging dogs reported as having barked (*n* = 26) was lower than that for dogs kept in kennels (*n* = 35; *p* = 0.002; Figure 5), whereas the proportion of free-ranging dogs reported as having growled (*n* = 20) was higher than that for dogs kept in kennels (*n* = 7; *p* = 0.003; Figure 5).

**Figure 6:**
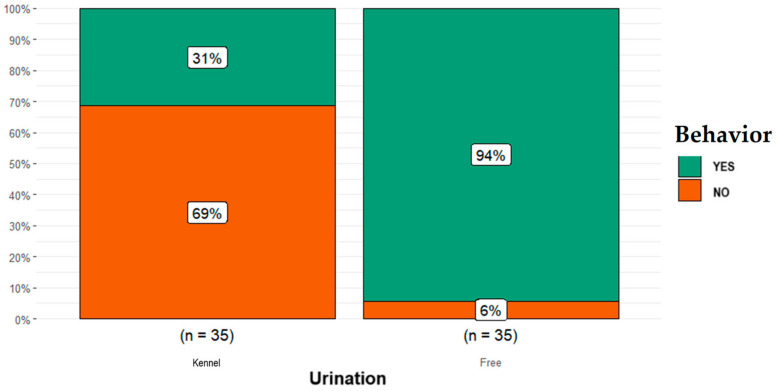
Differences obtained in the excretions category (i.e., dog marking with urine) between the free-ranging Fonni’s Dogs and those kept in kennels.

Concerning the excretions category, the proportion of free-ranging dogs reported as having marked with urine (*n* = 33) was higher than that for dogs kept in kennels (*n* = 11; *p* = 0.0001; Figure 6). 

No statistically significant differences in the other behaviors (i.e., scratching, sniffing, shivering, shaking, mouth shut, yawning, panting, licking, tail between the legs, wagging tail widely and quickly, turned forward ears, turned back ears, whining dog, marked with feces, gaze at owner, gaze at researcher, sniffing at researcher, contact with researcher) between the free-ranging Fonni’s Dogs and the Fonni’s Dogs kept in kennels were observed (Table 2).

## 4. Discussion

A deep understanding of canine communication in the form of visual, olfactory, and acoustic signals as well as of the other behavioral characteristics is of paramount importance to recognize the dog’s motivational state and its relationship with conspecifics and human beings [23,24,25].

The current study revealed, by detailed behavioral evaluation and statistical analysis, that several categories of the dogs’ body language herein investigated were associated with the living arrangements. Also, it might be where the dogs were observed that affected their behavior. Indeed, dogs of both groups were observed in their environment (kennel dogs in their kennel and free-ranging dogs on the farm) in which they were free to explore. It could be hypothesized that the behavior of dogs under the outdoor conditions may have been influenced by the presence of the stranger; however, both dogs in kennels and free-ranging dogs had limited contact with strangers because these dogs were used for flock defense on farms in which stranger humans are limited. Overall, the dogs subjected to the first condition (allocated in kennel) show common characteristics due to the confined environment: the hind limbs are under them, the body often seems to adapt to the terrain in which these dogs are forced to live, often near steep slopes and steep terrain. Indeed, though the kennels size were consistent with the animal’s ability to move freely, the Fonni’s Dogs were not free to explore the entire farm as is the habit of this breed. According to the second condition (free-ranging animals), dogs are an integral part of the community, they are free to move within the boundaries of the property and live with animals of different species. The role assumed by these dogs is that of guardians of the territory, and defenders of the herds. A lower percentage of ears turned back, and low posture was observed in free-ranging dogs compared to dogs kept in kennels, suggesting a dominance trait of free-ranging dogs. This dominant trait may have contributed to the differences found in the behaviors observed between free-ranging and kennels dog groups. A dominant dog shows a self-assured gait, a large, confident body posture, raised head, raised ears, larger pupils, curled lips, and carries the tail high with a slight wag [26]. The normal body language of a dog when greeting a human or another dog includes the movement of its ears up and down [26]. However, a forward ear position is associated with a state of heightened attention [27], motivation, confidence, and/or aggression, whereas a backward ear position is often associated with submission and/or fear [26]. The proportion of free-ranging dogs reported as having interacted with researcher (i.e., interaction by eyes, sniff and/or movement toward researcher) was lower than that for dogs kept in kennels, although statistical significance between the living conditions after Bonferroni’s correction test was lost. The recognition of specific odors and eye contact is essential for developing social skills [28]; however, in dogs, staring eye-to-eye means opposition [26]. Submissive dogs avoid direct eye contact with the dominant dog, and it has been suggested that the relative status of the dogs is determined by this visual communication [29]; this changes if the dog looks at a human. Indeed, the tendency of dogs to have periods of face/eye contact with people longer than socialized wolves was observed in the food task [30]. Thus, dogs do not watch other dogs’ eyes; however, it is thought that an effect of domestication is that dogs will watch a person’s eyes. Furthermore, while face/eye contact indicated the superior and inferior relationship between dogs [26], looking up at a human face and making eye contact can be categorized as body language focusing or expectation on a human. Overall, from the observation of all the enrolled animals, it emerged that both the dogs assigned to the kennels and the free ones showed a strong collaborative motivation with the owner. Noteworthy, this attitude could be exploited not only in guarding herds and property but also in socially useful functions including canine units of civil protection and law enforcement.

Fonni’s Dogs could be compared to other shepherd dog breeds due to the similar tasks and aptitude of these breeds. However, shepherd dogs have a predatory motivation channeled to the animals present in their territory and their work takes place mainly in solitary conditions. Shepherd dogs are linked more to the pastoral community but little to humans, they develop a temporary territoriality, and are not very trainable. Indeed, they do not respond promptly to commands and they do not have behavior related to predatory patterns. The Fonni’s Dogs distinguish between what belongs to the pastoral community and what is external, but they have a stronger relationship with humans [20]. While living in harmony with different species within the pastoral community, the predatory instinct is marked for everything that is not part of their community. They protect any animal that is part of their territory, but, if properly guided, they can be skilled hunters outside of this.

The character of many subjects belonging to this breed is traditionally considered as very aggressive and most of the breeders emphasize this characteristic as of great value. Worthy of note, the behavioral observation of Fonni’s Dogs herein investigated seems to confirm the proverbial aggression attributed to this breed, as highlighted by the high percentage of the dogs showing aggressive body language (i.e., the 60% of investigated dogs displayed a forward ear position, 38% of dogs growled, and 36% of dogs showed their teeth). The breed motivations (guarding and defense of the herds and territory) are satisfied both in kennels breeding and with animals free in the property. By studying the behavioral characteristics of these groups of dogs, we tried to understand what could be the causes of such coherence and communication abilitiy in dogs allocated in kennels, when we would have expected behaviors attributable to stress due to confinement. Probably, the behavioral balance of the Fonni’s Dogs under the two outdoor conditions could be attributed to correct communication between dogs and owners. As a matter of fact, the kennels were strategically positioned so that the view of the dogs was such that they could perform their function as guardian (sentinel) and the subsequent gratification on the part of the owner. According to the anthropologist Bandinu [31], the Sardinian pastoral culture is an archaic culture which has its social refinements, but which from the point of view of communication between bodies is very direct, cut on opposing sides (friend and foe), has no mediations, and is a decisive culture. Therefore, the relationship between the owners and the Fonni’s Dogs both confined in kennels and left free seems to be based on a deep mutual respect, on the coherence of behaviors, and on the construction of specific roles.

In the present study, a randomization was not carried out for the assignment of the dogs to the two groups, but we decided to divide the dogs according to their management conditions in order to avoid perturbation of their welfare status. Briefly, dogs who, prior to the study, lived in kennels were assigned to the kennel condition and dogs who, prior to the study, did not live in kennels were assigned to the free-ranging condition. This group assignment decision leads us to justify the differences found in the behaviors of the Fonni’s Dogs as associated with the two different living conditions and, therefore, the ‘baseline’ group differences found herein could be considered a limitation of the study. Moreover, the presence of a stranger, in the role of the dog’s behavior observer, could have influenced the behavior of dogs. Further studies; both involving a behavioral observer who has become familiar with the dogs which will be enrolled in the study, and assigning dogs that have always lived free in a group that is placed in kennels; are recommended in order to better define how the behavior of the Fonni’s Dog responds to a drastic change in its management.

## 5. Conclusions

In the dog’s body language, each emotion is expressed using the whole body. Thus, elucidation of the entire body language is worthy of investigation as it represents the key to understanding the level of motivation and attention as well as the dog’s state (including the friendly, playful, fearful, submissive, dominant, and aggressive states with conspecifics and humans). The present study showed for the first time the common social and communicative behaviors of Fonni’s Dogs, both kept in kennels and free ranging on the property, improving the scant knowledge currently available on the behavior features of this dog breed. The preliminary results that have been obtained here outline a behavior consistent with the attitude of the Fonni’s Dogs and highlight a good behavioral balance of this dog breed. Both the dogs confined into kennels and the free ones showed a strong collaborative motivation with the respective owner. Noteworthy, this attitude could be exploited not only in guarding herds and property but also in socially useful functions. The current study lays the foundations for a deeper investigation in this field in order to pave a way toward more informed and ethical Fonni’s Dog breeding.

## Figures and Tables

**Table 1:** Behaviors recorded from Fonni’s Dog during the examination and their definitions.

Observed Behavior	Description
Body expressions	
Scratching/Sniffing/Shivering/Shaking	The dog scratched itself/The dog sniffed the ground or straight ahead/The dog trembled/The dog shook
Low postures	The dog’s tail was lowered, its ears faced backwards, or its legs were bent; at least two of these postures were exhibited
Mouth expressions	
Mouth shut	The dog shuts its mouth
Yawning/Panting/Licking	The dog yawned/The dog panted/The dog licked its mouth
Lips curling	The dog’s mouth opens, the teeth are visible, and the cheek muscles look firm
Teeth showing	The dog showed front and back teeth, and the look is obviously aggressive
Tail language	
	The dog’s tail is between the legs
	The dog’s tail is up almost vertically
	The tail is not wagging
	The tail is wagging widely and quickly
Ear appearances	
	The dog’s ears are turned forward
	The dog’s ears are slightly turned back
	The dog’s ears are turned back
Vocalizations	
	The dog whined
	The dog barked
	The dog growled
Contact	
Gaze at owner	The dog gazed with its head oriented toward its owner
Gaze at researcher	The dog gazed with its head oriented toward researcher
Contact with researcher	The dog intentionally touched researcher
Sniff the researcher	The dog sniffed the researcher
Move to researcher	The dog moved toward researcher
Excretions	
Urination	The dog intentionally marks with urine
Defecation	The dog intentionally marks with feces

**Table 2:** Fisher’s exact test, following Bonferroni’s correction, results of no statistically significant differences in behaviors between the free-ranging Fonni’s Dogs and the Fonni’s Dogs kept in kennels.

Observed Behavior	Free Group	Box Group	*p*-Value
Body expressions			
Scratching/Sniffing/Shivering/Shaking	*n* = 0; 0%	*n* = 2; 2.86%	0.49
Mouth expressions			
Mouth shut	*n* = 4; 5.71%	*n* = 3; 4.28%	1.00
Yawning/Panting/Licking	*n* = 22; 31.43%	*n* = 20; 28.57%	0.80
Tail language			
Tail between the legs	*n* = 7; 10%	*n* = 9; 12.86%	0.77
Tail wagging widely and quickly	*n* = 19; 27.14%	*n* = 25; 35.71%	0.43
Ear appearances			
Ears turned forward	*n* = 20; 28.57%	*n* = 22; 31.42%	0.80
Ears slightly turned back	*n* = 24; 34.28%	*n* = 26; 37.14%	0.79
Vocalizations			
Whining dog	*n* = 14; 20%	*n* = 15; 21.42%	1.00
Excretions			
Marked with feces	*n* = 24; 35.71%	*n* = 15; 21.42%	0.03
Contact			
Gaze at owner	*n* = 15; 21.43%	*n* = 21; 30%	0.23
Gaze at researcher	*n* = 35; 50.00%	*n* = 29; 41.43%	0.03
Sniffing at researcher	*n* = 29; 41.43%	*n* = 19; 27.14%	0.02
Contact with researcher	*n* = 2; 2.86%	*n* = 5; 7.14%	0.43

## Data Availability

The data presented in this study are available on request from the corresponding author. The data are not publicly available due to privacy reasons.
